# Ultrasound-guided percutaneous needle electrolysis for musculoskeletal injuries: A scoping review considering implications for athletic populations

**DOI:** 10.17159/2078-516X/2026/v38i1a24229

**Published:** 2026-08-15

**Authors:** K Mashida, K Marsden, M Freschi, S Carmody

**Affiliations:** 1 Institute of Health Sciences Education (IHSE), Queen Mary University of London, London, United Kingdom; 2 Hull University Teaching Hospitals, Hull, United Kingdom; 3 Juventus F.C., Medical Department - Male First Team Club Doctor, Turin, Italy; 4 Amsterdam UMC location University of Amsterdam, Department of Orthopedic Surgery and Sports Medicine, Meibergdreef 9, Amsterdam, Netherlands

**Keywords:** Athletes, rehabilitation, ultrasound-guided intervention

## Abstract

**Background:**

Ultrasound-guided percutaneous needle electrolysis (PNE) is an emerging minimally invasive technique used for musculoskeletal (MSK) injury management. Despite its growing use in clinical practice, the literature reports mixed findings regarding efficacy, safety, and optimal application in athletic populations.

**Objectives:**

This scoping review examines the current literature on PNE for MSK injuries in athletes, evaluating its effectiveness in reducing pain, improving functional recovery, and enhancing return-to-play outcomes. The review also explores its safety profile and comparative advantages over conventional rehabilitation strategies.

**Methods:**

A systematic search was conducted in PubMed, Scopus, ScienceDirect, Google Scholar, and Web of Science. Peer-reviewed articles published between 2010 and 2024 were selected based on relevance to PNE, MSK injury management, and athlete populations. Studies were analysed for intervention protocols, outcome measures, and follow-up durations. Eligible articles included randomised controlled trials, cohort studies, pilot studies, systematic reviews, and meta-analyses. Data were charted for study design, population, intervention protocols, outcome measures, and follow-up duration. Findings were synthesised descriptively in line with a scoping review approach.

**Results:**

Eighteen studies met the inclusion criteria, encompassing randomised controlled trials, cohort studies, pilot studies, systematic reviews, and meta-analyses. Findings indicate that PNE significantly reduces pain and enhances functional recovery, particularly when combined with eccentric exercise. The most commonly treated conditions include patellar tendinopathy, supraspinatus tendinopathy, and lateral epicondylitis. While adverse effects are generally mild (e.g., transient soreness, hematoma), variations in treatment protocols hinder standardisation.

**Conclusion:**

PNE demonstrates promise as an effective treatment for MSK injuries in athletes, particularly when integrated with rehabilitation programs. However, methodological heterogeneity and limited long-term data necessitate further research to establish standardised protocols and optimise treatment outcomes. Expanding the evidence base through high-quality trials will be essential for its broader implementation in sports medicine.

Ultrasound-guided percutaneous needle electrolysis (PNE) has emerged as a novel technique for managing musculoskeletal (MSK) injuries, offering a minimally invasive approach that stimulates tissue repair and regeneration. The procedure involves the application of a high-intensity galvanic electrical current delivered through a fine needle under real-time ultrasound visualisation. This controlled microtrauma induces a localised inflammatory response, triggering a cascade of biological processes that promote tissue remodelling and recovery.^[1–3]^ Other commercial variations of this technique are marketed under names such as intratissue percutaneous electrolysis (EPI); however, the underlying principles are consistent.

Despite its relatively recent adoption, research has begun to elucidate the molecular mechanisms underlying PNE’s therapeutic effects, particularly in tendinopathies. Experimental animal studies have demonstrated that PNE activates key inflammatory pathways and enhances tissue repair.^[4]^ Abat et al. (2014)^[5]^ demonstrated that PNE modulates inflammation by upregulating anti-inflammatory proteins (PPAR-γ, VEGF) and suppressing degenerative markers (IL-1, TNF, COX-2). Galvanic current may also activate the NLRP3 inflammasome, triggering caspase-1–mediated release of IL-1β, a key cytokine in tissue repair and immune regulation.^[6]^ These initial findings provide a biological rationale for PNE’s application in managing MSK injuries.

One of the key advantages of ultrasound-guided PNE lies in its precision. Ultrasound guidance ensures accurate needle placement, significantly reducing the risk of complications compared to traditional palpation techniques.^[7]^ Figure 1 is a visual representation of the ultrasound-guided application of the needle to a localised injury. This precision is particularly critical in sports medicine, where optimal targeting of injured tissues is essential to maximise recovery and minimise downtime.

The efficacy of ultrasound-guided PNE has been demonstrated across various MSK conditions, particularly in tendinopathies, muscle injuries, and chronic pain syndromes.^[1,2, 5, 8–11]^

Meta-analyses have shown significant reductions in pain and improvements in functional outcomes when compared to conservative treatments.^[12]^ Combined approaches incorporating PNE, eccentric exercises, and structured rehabilitation programs have been particularly effective in expediting return-to-play timelines for athletes with injuries to the rectus femoris or adductor longus or with tendinopathies.^[13–15]^

In addition to its therapeutic potential, the safety profile of PNE has also been evaluated. Mild and transient adverse effects, such as local soreness and haematomas, are the most commonly reported complications, while serious complications associated with local application, such as syncope, skin lesions, nerve injuries, and metal allergies, are rare.^[16, 17]^

The aim of this scoping review is to evaluate the scientific literature on the use of PNE for managing MSK injuries, with a focus on its application in athletic populations. Specifically, the review explores the efficacy of PNE in treating a range of injuries, including tendinopathies, muscle and ligament injuries, and chronic soft tissue pathologies. Furthermore, the review examines the comparative advantages of ultrasound-guided PNE over conventional and emerging treatment modalities in terms of recovery time, pain reduction, functional improvement, and return-to-sport outcomes. Safety profiles, adverse events, and practical considerations are assessed to establish its relevance as a minimally invasive, low-downtime intervention for athletes.

## Methods

### Search strategy

This scoping review followed systematic search principles guided by the Preferred Reporting Items for Systematic Reviews and Meta-Analyses (PRISMA) framework. However, as the aim was to provide a broad qualitative synthesis of PNE for MSK injuries in athletes, formal risk-of-bias assessment, meta-analysis, and certainty grading were excluded.

A systematic literature search was conducted in PubMed, Scopus, ScienceDirect, and Web of Science to identify studies investigating ultrasound-guided percutaneous needle electrolysis (PNE) for musculoskeletal injuries. Searches were conducted between 1 October 2024 and 31 October 2024, with the final search performed on 15 May 2025.

**Fig. 2:**
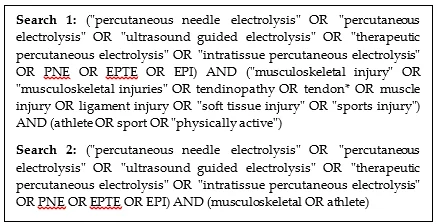
Search strings used to identify literature with potential relevance to the use of PNE for musculoskeletal injuries.

Filters were applied to limit the results to peer-reviewed articles on humans, published in English. Searches focused on studies published from 2010–2024, given the recent emergence of PNE as a clinical intervention. Search 1 was used to search the PubMed, Scopus, Science Direct, and Web of Science databases. Search 2 was used to identify literature from Google Scholar. Google Scholar was used as a supplementary search tool to identify additional relevant studies not indexed in traditional databases. Due to the large volume of results returned by Google Scholar, screening was limited to the first 200 results (10 pages) sorted by relevance. This approach is consistent with previous evidence-synthesis recommendations for the use of Google Scholar in systematic searching. The search strings used to identify the relevant literature are outlined below in Figure 2.

As PNE is an emerging therapeutic intervention with no standardised treatment protocol and is not appropriate for unsupervised use by the general public, consultation of the grey literature was not deemed appropriate.

### Eligibility criteria and study selection

This review focused on adults, particularly athletes, as the application of PNE in paediatric and adolescent populations remains underexplored. Initial exclusions included conference abstracts without linked published articles, non-English papers, and duplicates. Authors independently screened all records by title and abstract. Any discrepancies were resolved through discussion prior to the full-text screening stage.

The inclusion criteria were:

- Original research studies evaluating PNE for pain management, functional recovery, or rehabilitation outcomes.
- Studies reporting the use of PNE in clinical or athletic populations.
- Studies examining the physiological mechanisms or safety profile of PNE.
- Systematic reviews and meta-analyses, which were included specifically to map the current landscape of secondary evidence and aggregate broad findings.

The exclusion criteria were:

- Non-human or preclinical studies (unless specifically included for mechanistic context where relevant to PNE biological pathways).
- Paediatric or adolescent populations (<18 years), due to limited clinical application data in these groups.
- Non-English language publications; Conference abstracts without full peer-reviewed publications.
- Narrative reviews (non-systematic), editorials, opinion pieces, or commentary articles (though reference lists were screened for eligible primary studies).
- Studies not involving percutaneous needle electrolysis (PNE) or where PNE outcomes could not be isolated from other interventions; Studies not reporting clinical, functional, mechanistic, or safety outcomes related to musculoskeletal injury management.
- Duplicate publications or datasets (most complete or most recent version retained) or Studies where full text was not accessible after reasonable search effort.

The reference lists of all selected articles were reviewed to identify additional relevant studies that may have been missed during the electronic search. Additional contextual literature was considered in the Discussion to support interpretation of findings but was not included in the formal data extraction or synthesis.

### Data collection process

The following data items were extracted and tabulated by the reviewers: study design, author details, sample characteristics, intervention details (PNE protocols, ultrasound guidance, use of adjunct therapies), analytical strategies, and key findings related to pain, function, and safety. Any inconsistencies or discrepancies in data extraction were resolved by consensus through discussion among the authors.

### Quality control and data synthesis

All co-authors reviewed the data for accuracy and consistency. Studies were appraised for quality using criteria including sample size, methodological rigour, and clarity of treatment reporting. A scoping synthesis was then conducted to identify trends, gaps, and key insights into the efficacy, safety, and clinical application of PNE in athletes.

### Synthesis of results

Data were synthesised by outcome category - pain reduction, functional improvement, and safety profile. Consistencies and protocol variations were noted, with dosing and intensity data used to identify gaps in practice. Observational findings established preliminary benchmarks for PNE’s effectiveness, while controlled trials were examined to compare its efficacy with dry needling and conventional rehabilitation. No formal risk-of-bias tool, meta-analysis or certainty grading was undertaken; findings were synthesised descriptively and interpreted in light of study design, sample size, comparator and reporting limitations.

## Results

### Study design and participants

**Fig. 3:**
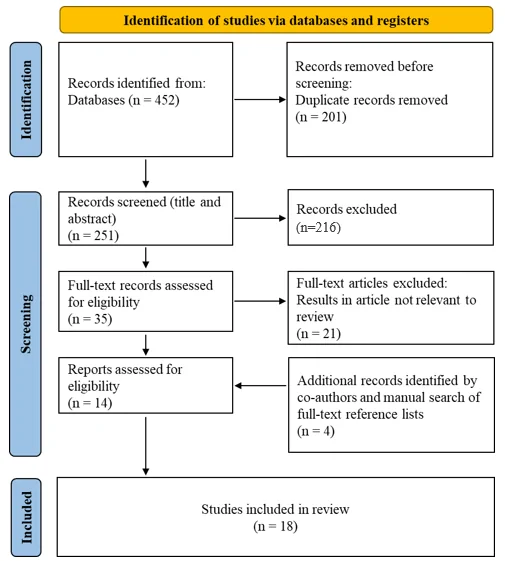
Flow of identification, screening, eligibility, and inclusion phases for the literature review.

Published literature was screened using the inclusion and exclusion criteria. Figure 3 presents a PRISMA-inspired flowchart documenting the outcomes at each stage of the search process. A total of 18 studies met the inclusion criteria and reported on the use of PNE as a treatment modality. The included studies comprised a systematic review (n=1)^[1]^, a meta-analysis (n=1)^[11]^, randomised controlled trials (RCTs) (n=7)^[9, 10, 18–22]^, quasi-experimental design (QED) studies (n=6)^[5, 23–27]^, a cohort study (n=1)^[15]^, a safety study (n=1)^[16]^, and an adverse effects study (n=1).^[17]^

The study populations varied, with eleven studies focusing on the general population and seven specifically investigating athletes. Using the Participant Classification Framework^[28]^, the athletic populations ranged from Tier 1/2 – Recreationally Active/Trained^[5, 24, 25, 29]^ to Tier 3/4 – Highly Trained/Elite.^[5, 15, 26]^ However, precise classification was challenging for some studies due to insufficient details on participants’ training regimens and competitive levels. Tables 1 and 2 categorise the included studies by pathology type and provide details on population characteristics, intervention protocols, outcome measures, follow-up duration, and key findings.

Various pathologies affecting both the upper and lower limbs were examined, with tendinopathies being the most frequently studied condition (n=10).^[5, 9, 17–23, 27]^

Patellar tendinopathy was the most common pathology (n=3)^[5, 18, 20]^, followed by supraspinatus tendinopathy (n=2)^[19, 21]^ and subacromial pain syndrome (n=1).^[9]^ Other conditions investigated included soleus (n=2)^[10, 25]^ and adductor longus injuries (n=1)^[26]^, lateral epicondylitis (n=2)^[22, 27]^, and Achilles tendinopathy (n=1).^[23]^

### Study protocols and PNE parameters

There was considerable variability in study protocols and treatment parameters across the included studies. Seven studies assessed PNE as a standalone intervention. The majority of studies explored PNE in combination with adjunct therapies, predominantly exercise-based interventions. Eight studies compared PNE with alternative treatments, such as exercise, dry needling, or sham interventions (e.g., ultrasound-guided needle insertion without the application of a galvanic current) ^[18, 20, 25]^, while five studies examined PNE alongside exercise as an adjunct therapy.

The specific PNE application protocols varied significantly across studies. Table 3 demonstrates the variation of approaches, with treatment frequencies ranging from single session interventions^[15, 26]^, to repeated therapy either biweekly^[18, 20, 29]^, or weekly.^[5, 9, 10, 19, 21–23, 25, 27]^

Current intensity settings varied, including low-current, prolonged applications (0.35 mA for 72 s^[9, 19, 21, 22]^), to higher level charge and time (2 mA for 3 s^[15, 26]^, 2.5 mA for 3 s^[10]^, 3 mA for 3^[18, 20, 29]^ or 10 s^[23]^, 5 mA for 3 s^[27]^). Needle diameters ranged from 0.25 mm^[18, 20, 29]^ to 0.3 mm.^[5, 9, 10, 15, 19, 22, 23, 25–27]^

Evidence from a murine animal model suggests that current intensities exceeding 6 mA may result in "electrolysis overdose," potentially leading to extensive tissue necrosis. This study presented evidence that 2 doses of galvanic current delivered at either 3 mA or 6 mA for 6 seconds did not compromise cell viability yet were able to elucidate an inflammatory response. In contrast, the 12-mA dose, administered using the same 2-dose regimen for 6 seconds, resulted in significant cell death, independent of the inflammasome. The rate of cell death was further exacerbated by increasing duration and overall number of galvanic current deliveries. In a clinical setting, the dose-response relationship between the galvanic current and localised tissue necrosis should be considered as part of the therapy's benefit-risk profile, given the potential risks associated with prolonged high-intensity doses or repeated applications.^[6]^

### Outcome measures

The primary outcome measures across studies included pain reduction, functional improvements, and structural changes. Pain reduction was the most commonly reported outcome (85% of studies), assessed primarily using the Visual Analogue Scale (VAS) and Numeric Pain Rating Scale (NPRS). Other validated scales for assessment were used for specific injuries such as VISA–P (Patellar tendon injuries), VISA–A (Achilles tendon injuries), DASH, and SPADI (Upper limb injuries), FAAM and DROM (Foot and Ankle injuries).

Studies focusing on athletic populations incorporated performance-related assessments, including Return to Play (RTP), Return to Training (RTT), GPS on-pitch monitoring, muscle endurance testing, and functional testing. Ultrasound imaging was utilised in the studies concerned with structural changes.

The follow-up period varied across studies, categorised into short-term (<1 month), mid-term (1–3 months) and long-term (>3 months). Only one study included all three follow-up durations. Seventy-two per cent included short-term followup, 28% included mid-term follow-up, and 33% included long-term follow-up.

### Effects of PNE on different pathologies

**Fig. 4:**
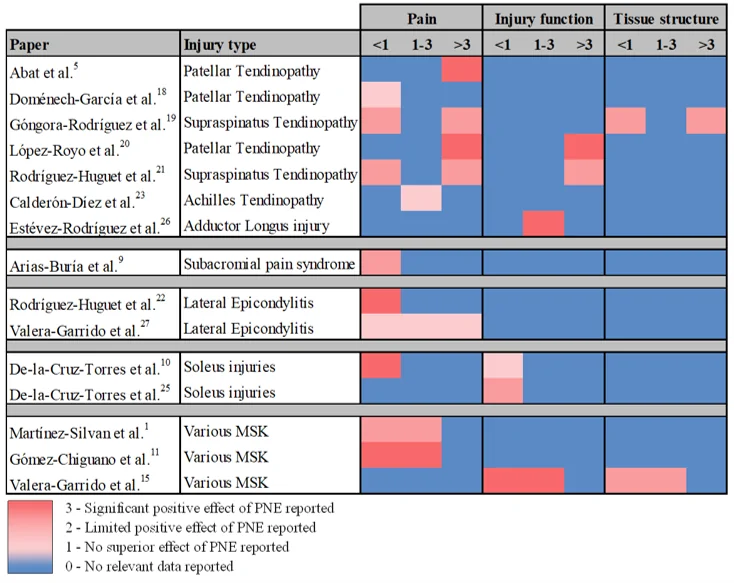
A heat map of the included studies grouped by primary pathology with defined endpoints; short-term (<1 month); mid-term (1–3 months); long-term (>3 months).

To facilitate comparison, data from the reviewed studies were visualised in a heat map (Figure 4). The studies were grouped by primary pathology and evaluated for their effects on pain reduction, functional outcomes, and tissue structure over short-, mid-, and long-term follow-up periods. The heat map represents descriptive categorisation of how outcomes were reported within each study. If a study did not describe any data relating PNE to a given endpoint, this was categorised as “no relevant data reported.” If a study described no benefit of PNE relative to comparators, this was categorised as “no superior effect of PNE reported.” The studies that described limited effects were categorised as “limited positive effect of PNE reported.” Finally, the studies that described a clear positive effect of PNE were categorised as “Significant positive effect of PNE reported.” Among studies reporting PNE outcomes, 10 studies examined tendinopathies (Achilles tendinopathy, Patellar tendinopathy, Supraspinatus tendinopathy, Subacromial pain syndrome, lateral epicondylitis), and 5 studies explored the effect of PNE on various other musculoskeletal injuries.

Outcomes related to pain, injury function, and tissue structure were reported across short (<1 month), mid-term (1–3 months), and long-term (> 3 months) follow-up periods. Pain outcomes were the most commonly reported endpoint. In studies investigating tendinopathy, several reported improvements in pain following PNE, mostly at short- and mid-term follow-up. The strongest reported positive effects at the long-term follow-up were observed in the studies concerning patellar tendinopathies. At the short-term followup, the strongest positive effect was seen in studies involving lateral epicondylitis and subacromial pain syndrome. Injury function was the second most reported outcome. Positive effects of PNE were observed in a small number of studies involving patellar tendinopathy, supraspinatus tendinopathy, and a mixed MSK cohort. Functional outcomes were frequently unreported in the remaining studies, which limits cross-study comparison.

Tissue structure was the least commonly reported outcome. Only a small number of studies reported structural changes following application of PNE. The findings suggested a limited positive effect at mid- to long-term follow-up in a mixed MSK injury population. A study by Góngora-Rodríguez J. et al., 2024^[19]^ investigating the combination of PNE, percutaneous peripheral nerve stimulation, and eccentric exercise showed both significant within- and between-group structural improvements. The within-group changes suggested that structural improvement was achieved across all the treatment modalities. However, the between-group differences favoured the combination of treatments including PNE, demonstrating improvements across the parameters for structural change (reduction in echogenicity, thickness, and hypervascularisation; P<0.05, P<0.001, P<0.001, respectively). The majority of studies, however, did not include structural imaging, which meant this outcome was not able to be consistently assessed.

Across the reported outcome domains, most reported outcomes were seen at the short-term follow-up, with fewer studies reporting outcomes at the mid-term and long-term endpoints. These findings were further organised in Figure 4, which provides a visual comparison of PNE’s effects across different MSK conditions and treatment endpoints. The colour gradient reflects the descriptive outcomes and does not represent statistical analysis of the outcomes.

### Comparison of PNE with other treatment modalities

**Fig. 5:**
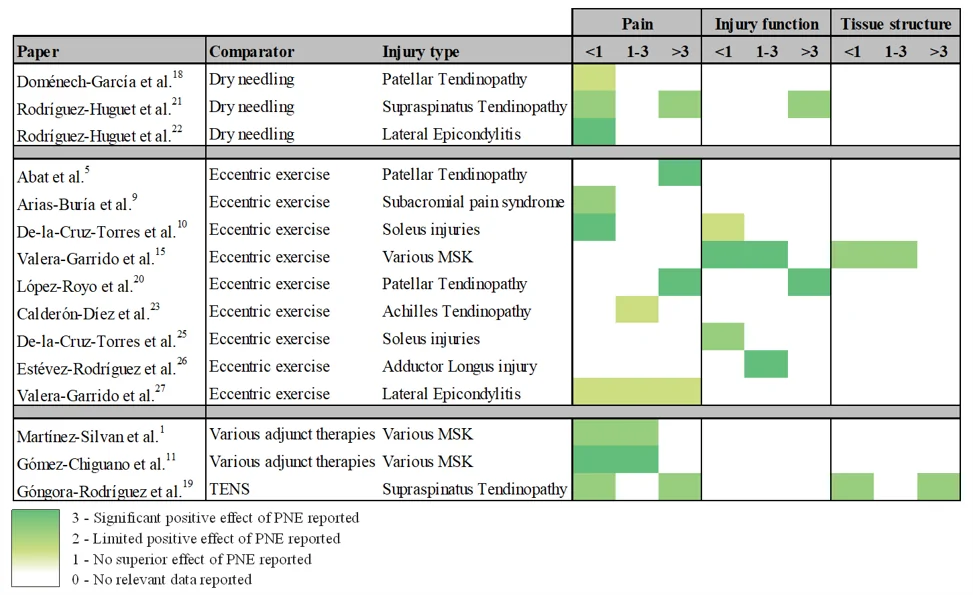
A heat map of the included studies grouped by comparator, with defined endpoints: short-term (<1 month), mid-term (1–3 months) and long-term (>3 months). MSK, musculoskeletal; PNE, percutaneous needle electrolysis; TENS, transcutaneous electrical nerve stimulation.

PNE has been evaluated against various treatment modalities including exercise programs, dry needling, and injections of corticosteroids or platelet-rich plasma (PRP). The effectiveness of PNE has been assessed at different time points, with some studies focusing on short-term effects (<1 month), while others have examined medium-term (1–3 months) and long-term (>3 months) outcomes. Figure 5 provides a visual representation of these findings, grouped by comparator.

Short-term improvements in pain scores were observed in patients treated with dry needling.^[18,20,21,22,30]^ One study investigating supraspinatus tendinopathy reported sustained long-term improvements in pain and injury function following dry needling.^[21]^ Patients receiving eccentric exercise alongside PNE showed improvements in pain across short-, medium-and long-term follow-up periods.^[5,11,13,20,21,27,30]^ Studies also reported enhanced functional recovery in athletes and general MSK injury^[10, 11, 30]^ when exercise regimens were combined with PNE. Structural improvements in tendon or muscle integrity were observed in one study assessing tissue remodelling following PNE intervention.^[15]^

## Discussion

### Application of PNE in MSK injuries

#### Pain management

A randomised controlled trial (RCT) by De-la-Cruz-Torres et al. (2020)^[10]^ evaluated the efficacy of PNE combined with eccentric exercise for chronic soleus injuries in dancers—an overuse condition of the deep calf muscle caused by repetitive strain and biomechanical overload. The combination therapy produced the greatest pain reduction (NPRS: Δ53.6%) compared with PNE or exercise alone. Similarly, a pilot study by De-la-Cruz-Torres et al. (2022)^[25]^ assessed ultrasound-guided PNE with therapeutic exercise in female soccer players and found reductions in palpation and stretch-induced pain, though no significant difference compared with sham needling. Both studies, however, were limited by small sample sizes, narrow populations, and predominantly female cohorts, restricting generalisability to broader athletic groups. Arias-Buría et al. (2015)^[9]^ assessed the use of PNE combined with eccentric exercises to manage subacromial pain syndrome (SAPS). Participants receiving PNE reported significantly greater pain reductions (NPRS: Δ5.6) compared to the exercise-only group (Δ3.7), exceeding the minimal clinically important difference (MCID). The MCID represents an alternative patient-focused endpoint which denotes the smallest change in the treatment outcome, in this case pain reduction, that a patient would perceive as beneficial. Improvements in the disability (DASH) scores were also observed but fell below the MCID threshold, highlighting the need for further investigation. The short-term follow-up (1-week post-intervention) and lack of a no-treatment control group limit the ability to attribute improvements solely to the intervention. Additionally, the potential placebo effects of advanced technologies such as ultrasound guidance remain unexplored. Valera-Garrido et al. (2014)^[27]^ reported significant pain reduction in lateral epicondylitis using a multimodal approach combining PNE, eccentric exercise, and stretching, with VAS scores falling from 60.2 to 6.0 and 86% of participants pain-free at discharge. Ultrasound showed improved tendon hypoechogenicity and hypervascularity, and benefits persisted at 26- and 52-week follow-up, though the lack of a control group limits interpretation. In non-elite athletes with patellar tendinopathy, López-Royo et al. (2021)^[20]^ compared PNE, dry needling, and sham needling, each combined with eccentric exercise. All groups showed significant pain reduction (VAS), but no between-group differences, suggesting exercise may drive much of the improvement. This highlights a broader trend in the literature: while some studies suggest PNE superiority, others show no significant between-group differences compared with active controls. These conflicting results are likely driven by significant methodological heterogeneity. Furthermore, even where superiority over dry needling or exercise is suggested, confidence intervals frequently overlap, and the between-group differences do not consistently exceed MCID thresholds. A systematic review by Martínez-Silvan et al. (2022)^[1]^ found moderate short- to mid-term pain relief with PNE across 21 studies, though 62% of studies were found to have moderate-to-high bias, underscoring the need for higher-quality evidence.

Rodríguez-Huguet et al. (2020)^[21]^ compared PNE and trigger-point dry needling in a single-blinded RCT for supraspinatus tendinopathy, with both groups performing eccentric exercises. PNE achieved greater and longer-lasting pain reduction over one year illustrated with the NPRS (mean difference of −2.24, 95% CI: −3.33 to −1.16 and P<0.001), likely through its localized inflammatory response that promotes tissue repair and complements eccentric loading. However, the small sample size limits generalisability. Additionally, despite the statistically significant results no MCID was reported for pain. Similarly, Valera-Calero et al. (2021)^[30]^ compared high-intensity PNE (HIPE) and low-intensity PNE (LIPE) with dry needling in patellofemoral pain syndrome. All treatments reduced pain, but PNE showed superior short-term outcomes, possibly due to its combined mechanical and electrical effects on nociceptive inputs. This study reported several 95% confidence intervals for between-group comparisons. Most of the reported 95% confidence intervals were wide and overlapped, indicating no significant differences between the groups for the following: pain pressure thresholds at the myofascial trigger point; high vs low intensity PNE; high intensity vs dry needling; and low intensity vs dry needling. The only statistically significant comparison was between PNE and dry needling. PNE was found to be less painful than dry needling (mean difference of 1.8, 95% CI −3.2 to −0.4). The study did not report any MCID thresholds, making it difficult to draw conclusions from the results.

PNE’s role in pain management is also influenced by psychological factors, as shown by Doménech-García et al. (2024).^[18]^ This study highlighted the placebo and nocebo effects associated with needle-based interventions. Patient expectations, previous experiences, and therapeutic context, as well as the manner in which the procedure is explained, can significantly influence pain perception. This underscores the importance of clinician-patient communication in optimising treatment experiences, particularly for athletes who may have heightened recovery expectations. Notably, studies have shown that negative expectations can elicit nocebo responses, leading to increased pain after needling, even when the intervention is a sham.^[31, 32]^ One investigation found heightened mechanical hyperalgesia following sham dry needling in individuals with chronic shoulder pain, attributing this to nocebo effects induced by pre-treatment instructions.^[33]^ Such findings underscore the powerful influence of contextual and subjective factors on outcomes. Moreover, demand characteristics may also skew results if participants infer what the evaluator expects, especially in unblinded or single-blind settings. The absence of psychological measures such as fear, emotional stress, or pain catastrophizing limits attribution of outcomes solely to the physiological effects of PNE. Including these variables in future studies would provide greater clarity on the relationship of experienced pain and the application of PNE.

#### Functional improvements

In addition to pain relief, functional improvements are a critical outcome in the management of musculoskeletal (MSK) injuries, especially for athletes aiming to return to pre-injury performance levels. De-la-Cruz-Torres et al. (2020)^[10]^ reported significant improvements in dorsiflexion range of motion (DROM) and functional outcomes among dancers with chronic soleus injuries. The significant differences (P < 0.05) between pretreatment and posttreatment Dance Functional Outcome Survey (DFOS) scores demonstrated the observed improvements in functionality. The group receiving combined PNE and eccentric exercises achieved superior results compared to groups receiving either intervention alone. When US-guided PNE was used in combination with therapeutic exercises amongst female soccer players with soleus injuries, improvements were observed in satisfaction levels and DROM.^[25]^ However, the study found no significant impact of the combined therapy on soccer performance outcomes, as measured by the curve sprint test, raising questions about the intervention's efficacy in enhancing athletic performance. Furthermore, while the study reports high satisfaction among participants with the treatment, it does not provide a detailed analysis of long-term outcomes or potential adverse effects associated with PNE. Valera-Garrido et al. (2020)^[15]^ investigated PNE combined with a rehabilitation program in professional male soccer players with rectus femoris injuries. The study utilised load monitoring via GPS and accelerometers, as well as individualised drills, to restore neuromuscular function. Specifically, GPS tracking of variables such as high-intensity running, sprint velocities, and peak acceleration allowed clinicians to compare post-injury performance directly against the players' pre-injury baselines. This quantitative approach ensured athletes met objective mechanical thresholds before being cleared for RTT—the resumption of full team practices—and RTP—the return to competitive matches. By individualising recovery through this data, players achieved a rapid average RTT of 15.6 days and RTP of 20.1 days, with zero reinjuries reported over a 20-week follow-up. Concomitant use of quantitative data-capture methodologies in specific athlete populations will allow the sub-classification of injury groups by mechanism of injury. By improving the understanding of the mechanism of injury, the specific pathologies which might be best suited to PNE-centric interventions can be identified. Estévez-Rodríguez et al. (2024)^[26]^ later followed the same treatment protocol as proposed by Valera-Garrido et al. (2020)^[15]^ and reported no recurrence of injury at their final follow-up at eight weeks, compared with the epidemiological average of 14%. This longterm benefit of PNE on functional recovery is supported by Rodríguez-Huguet et al. (2020a)^[21]^, who observed sustained improvements in joint function over a one-year follow-up in patients treated with PNE for supraspinatus tendinopathy. Calderón-Díez et al. (2020)^[23]^ specifically examined the role of PNE in chronic Achilles tendinopathy when combined with eccentric exercises. Functional measures, such as the VISA-A score, demonstrated significant improvements. Due to limitations in the evidence presented by these papers, including selection bias in small groups and, in some cases, a lack of substantial controls, the proposed improvements in overall recovery and return to play should be considered as hypothesis-generating for future research. Similar findings in the context of patellar tendinopathy ^[5]^ highlighted notable improvements in functional performance, as measured by the VISA-patella tendon (VISA-P) score. The integration of PNE with eccentric exercise facilitated athletes’ return to pre-injury activity levels within a relatively short period. Another study focused on patellar tendinopathy; López-Royo et al. (2021)^[20]^ reported short-term improvements in quality of life at 10 weeks following PNE combined with eccentric exercise; however, these benefits were not maintained at long-term follow-up. Góngora-Rodríguez et al. (2024)^[19]^ provided further evidence of PNE’s functional benefits in athletes with supraspinatus tendinopathy when used with exercise. Their study demonstrated significant improvements in strength, electromyographic activity, and tendon characteristics (e.g., echogenicity and hypervascularization) when PNE was combined with eccentric exercise. However, the combination of therapies introduces confounding variables, making it challenging to isolate the specific contribution of PNE to functional recovery. Functional improvement comparisons have also been made between PNE and dry needling.

Valera-Calero et al. (2021)^[30]^ compared functional outcomes between PNE and dry needling for patellofemoral pain syndrome (PFPS). Both interventions resulted in functional improvements, though the authors noted that evidence remains inconclusive, as some studies found no significant differences between the two treatments. An RCT comparing PNE with dry needling for lateral epicondylitis (LE) found that both groups experienced significant functional gains, with improvements sustained over time.^[22]^ These results align with a previous study which indicated that PNE enhances functional recovery, particularly when combined with active rehabilitation strategies.^[22]^ However, across these trials methodological heterogeneity is high, and between-group differences often fail to reach clinical significance or display overlapping confidence intervals. The small sample sizes and limited functional outcome measures highlight the need for further investigation to solidify whether PNE offers a true distinct advantage over comparators, particularly those including physical activity.

A meta-analysis by Gómez-Chiguano et al. (2021)^[11]^ identified significant functional improvements associated with PNE across various MSK conditions, including non-specific shoulder pain and patellar tendinopathy. However, the review emphasized the need for more robust RCTs to isolate the effects of PNE from cointerventions like exercise. However, Martínez-Silvan et al. (2022)^[1]^ found inconsistent improvements in injury-related function across studies.

#### Safety and adverse events

PNE is generally well tolerated for MSK injuries, though minor risks exist. Margalef et al. (2021)^[16]^ reported that variations in needle composition, morphology, and resistance can alter electrical conductivity and cause localized temperature rises, potentially leading to tissue damage if not properly controlled. This is particularly significant for athletes, whose injuries often involve high tissue sensitivity. A lack of standardised protocols can result in mild adverse effects such as hematoma or transient local pain.^[2, 16, 17]^ Overall, when applied appropriately, PNE shows a favourable safety profile, with reactions such as soreness resolving within 36 hours and short-lived delayed-onset muscle soreness (DOMS) during the first treatment week.^[9]^

De-la-Cruz-Torres et al. (2016)^[24]^ observed that applying PNE to the patellar tendon in healthy male footballers elicited autonomic responses, including increased parasympathetic activity, raising concerns about potential vasovagal reactions during treatment. Although these findings underscore the need for monitoring autonomic responses, the study’s homogenous sample limits generalisability to female athletes or those with comorbidities, and the mechanisms underlying these effects remain unclear. Simple measures such as screening for previous vasovagal events or positioning the patient in a supine position could help mitigate such reactions.

A systematic review by Fakontis et al. (2023)^[34]^ comparing PNE with dry needling found a statistically significant advantage for PNE in pain reduction; however, the clinical relevance was uncertain as improvements rarely exceeded the MCID. High risks of bias, particularly in blinding, and heterogeneity across study designs further limit confidence in these findings.

### Implications for practice

#### Which types of injuries or athlete populations may benefit most?

Used in sports medicine, PNE has proven to be a versatile and effective treatment for athletes with tendinopathies of the Achilles^[23]^ and patellar tendons.^[5,11,20]^ Through the mechanism of collagen regeneration and phagocytic facilitation, PNE contributes to the regenerative processes of soft tissue ECMs.^[5]^ Professional football players and dancers have also experienced faster recovery and reduced return-to-play time with this treatment.^[10,15,26]^ Both elite and recreational athletes could benefit from the incorporation of PNE into the recovery pathways due to the reduction in pain and recovery time, however due to the potential for mild adverse events, it is more likely to be used in a professional sports setting where the benefit-risk profile would be most favourable, and where clinicians are present to monitor for adverse effects. For athletic populations, where minimal downtime is critical, ultrasound guidance and precise parameter control are essential to mitigate risks such as hematoma, autonomic imbalance, or thermal injury. Reported adverse effects are typically mild and transient, reinforcing the importance of practitioner expertise and individualised treatment planning to ensure both safety and efficacy.

#### Is PNE superior to alternative treatment modalities?

Evidence comparing PNE with alternative interventions, such as corticosteroid or platelet-rich plasma (PRP) injections, as well as manual therapy, is limited and largely found in studies outside the formal scope of this review. Available evidence suggests that PNE may have a more favourable adverse event profile than injection-based therapies.^[36, 37]^ A small study comparing PNE with manual therapy for upper extremity injuries reported comparable short-term outcomes, with some improvements favouring PNE at three months based on DASH and SPADI scores.^[38]^ However, these differences should be interpreted with caution and through a contextual lens, given the small sample size, potential overlap in effect estimates, and uncertainty about whether changes exceeded the MCID thresholds.

#### Future directions

Future research on PNE should aim to refine treatment protocols by clarifying optimal dose, frequency, and duration across different tissue types. The collagen ultrastructure varies markedly between tendons, ligaments, and muscles: tendons and ligaments are predominantly composed of type I collagen, arranged in tightly wound fibrils, though tendons contain a higher proportion (95–99%) than ligaments (70–80%).^[39]^ In contrast, muscle tissue contains much less collagen, which is distributed mainly to the extracellular matrix and surrounding connective tissues.^[40]^ Since PNE exerts its therapeutic effects partly through collagen recruitment and extracellular matrix remodelling^[5]^, these structural differences may influence treatment responsiveness. Given the narrow therapeutic window reported (1–8 mA)^[5, 9, 14]^, denser tissues may require adjustments in dosage or treatment frequency to achieve optimal outcomes.

Although current literature does not provide consistent empirical support, there is growing clinical interest in ultrasound-guided PNE for chronic conditions associated with impaired tissue mobility, particularly restricted fascial or limb sliding due to scar tissue or nerve entrapment from adjacent fibrosis. Preliminary evidence demonstrated positive outcomes with PNE in treating sciatic nerve entrapment in a murine model.^[41]^ Anecdotal clinical observations also suggest potential benefits in targeting interfaces that limit soft tissue gliding, with the galvanic current acting as an advanced form of fibrolysis when combined with ultrasound precision. While these findings are encouraging, they remain unvalidated by controlled animal or microscopic studies and should therefore be viewed as a hypothesis to be tested by future work. This emerging therapeutic avenue warrants systematic investigation to elucidate its mechanisms, efficacy, and possible role in managing chronic soft-tissue restrictions.

### Limitations

This review underscores the potential of ultrasound-guided PNE as a treatment for musculoskeletal injuries in athletic populations. However, several limitations in the existing literature limit the strength of the current evidence and hinder the development of standardised clinical protocols.

A major limitation is the heterogeneity of study designs and methodologies. Substantial variation in PNE parameters, such as current intensity, treatment duration, and frequency, complicates comparisons across studies and prevents the establishment of optimal dosing guidelines. Furthermore, many investigations have small sample sizes or focus on niche athletic groups, limiting the generalisability of the results to broader sporting and non-athletic populations.

Most available studies report only short-term outcomes. Although improvements in pain and function are consistently demonstrated, evidence for long-term efficacy, recurrence rates, and the cumulative impact of repeated treatments remains scarce.

Finally, methodological weaknesses, such as limited blinding, the absence of appropriate control groups, and a moderate-to-high risk of bias, further constrain the validity of the reported findings. These issues highlight the need for large-scale, well-designed clinical trials to determine PNE’s comparative effectiveness and long-term safety relative to established interventions such as dry needling or platelet-rich plasma therapy.

## Conclusion

Ultrasound-guided percutaneous needle electrolysis (PNE) is an emerging modality with potential benefits for managing MSK injuries in athletes. It offers a minimally invasive approach that combines precision with efficacy, demonstrating significant short-term benefits in pain reduction and functional improvement. When integrated with eccentric exercise and structured rehabilitation programs, PNE appears to enhance recovery timelines and support a safe return to play.

Despite these advantages, the current evidence base remains insufficient to establish standardised treatment protocols or to fully evaluate long-term outcomes. Addressing the methodological and population-based limitations identified in this review is crucial to refining PNE’s role in sports medicine. Future research should focus on larger, more diverse populations, standardised protocols, and extended follow-up periods to better understand the technique’s efficacy, safety, and optimal applications.

While several studies report favourable outcomes for PNE, these findings should be interpreted with caution. Across the included literature, improvements in pain and function are frequently observed within PNE groups; however, between-group superiority over comparator interventions is inconsistent. In several trials, including those comparing PNE with dry needling, eccentric exercise, or sham interventions, no statistically significant between-group differences were observed despite meaningful within-group improvements.

Where PNE demonstrated apparent superiority, effect sizes were often modest, confidence intervals overlapped between groups, or improvements did not consistently exceed established MCID thresholds. This suggests that while PNE may contribute to clinical improvement, its incremental benefit over active comparators remains uncertain.

Due to the significant methodological and population heterogeneity in the reviewed literature, it is not possible to draw definitive conclusions regarding the overall superiority of PNE. Further large-scale studies would aid in identifying the specific groups or pathologies that may benefit most from PNE. Collectively, the evidence supports a potential role for PNE as part of a multimodal rehabilitation strategy rather than as a clearly superior standalone intervention.

## Figures and Tables

**Fig. 1:**
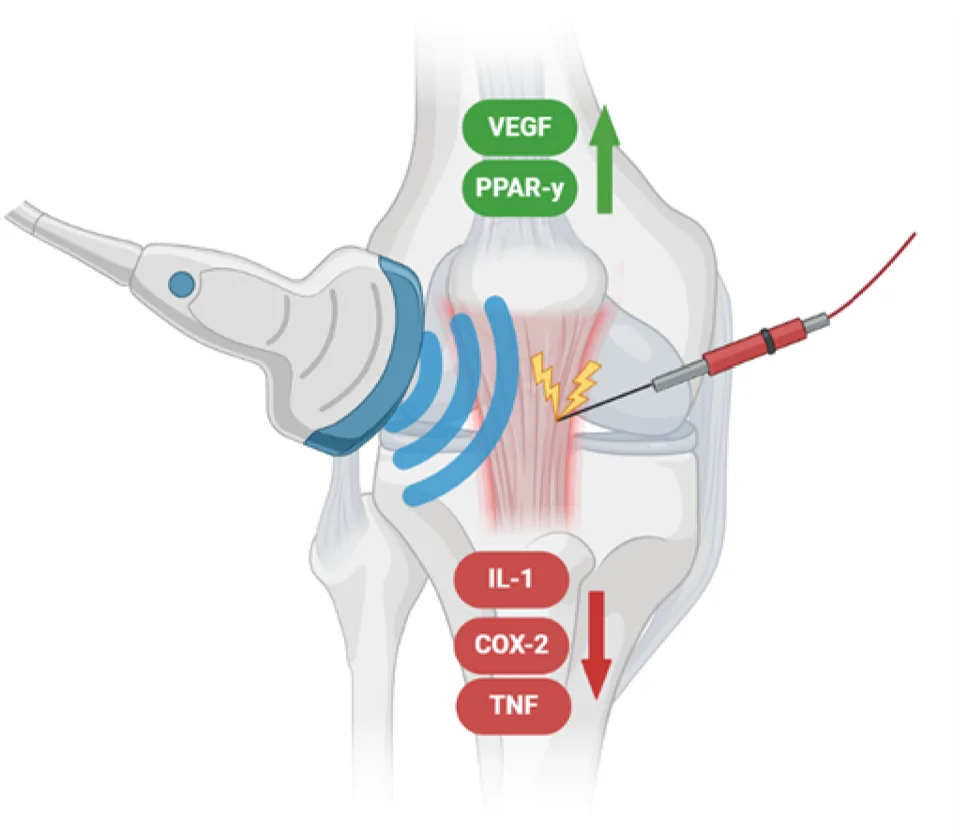
Needle approach during the application of ultrasound-guided percutaneous needle electrolysis (PNE). US, ultrasound; VEGF, vascular endothelial growth factor; PPAR-γ, peroxisome proliferator-activated receptor gamma; IL-1, interleukin-1; COX-2, cyclooxygenase-2; TNF, tumour necrosis factor.

**Table 1:** Reviewed studies focusing on tendinopathies, providing details on population characteristics, intervention protocols, outcome measures, follow-up duration, and reported effects

Study population	Sport	Injury/condition	Study design	Intervention	Outcome measures	Follow up	Reported effect	Study limitations
**Abat F, et al. 2014** ^[5]^								
Physically active adults	Running, football, basketball, volleyball	Patellar tendinopathy	Prospective case series	Ultrasound-guided EPI + eccentric exercise	VISA-P, pain, function	Short-and midterm	Improved pain and VISA-P	No control group, no blinding, single centre
**Abat F, et al. 2015** ^[13]^								
Physically active adults	Running, football, basketball, volleyball	Patellar tendinopathy	Prospective observational study	EPI + eccentric exercise	VISA-P, pain, function	Short-and midterm	Improved outcome	No randomisation, no control, possible selection bias
**Arias-Buría JL, et al. 2015** ^[9]^								
Adults with subacromial pain	None	Subacromial pain syndrome	Randomised controlled trial	PNE + exercise vs exercise	Pain, DASH, SPADI	Short-and midterm	PNE improved outcomes	Limited blinding, single centre, modest sample size
**Calderón-Díez L, et al. 2020** ^[23]^								
Adults with Achilles tendinopathy	Running, football, jumping sports	Chronic Achilles tendinopathy	Prospective cohort study	PNE + eccentric exercise	VISA-A, Pain	Short-and midterm	Improved outcomes	No comparator, no blinding, single centre
**Doménech-García V, et al. 2024** ^[18]^								
Adults with patellar tendinopathy	Running, football, basketball, volleyball	Patellar tendinopathy	Randomised controlled trial	Expectation-modulated PNE	Pain, VISA-P	Short-term	Expectation influenced outcomes	Limited sample size, short follow-up, complex contextual factors
**Góngora-Rodríguez J, et al. 2024** ^[19]^								
Adults with supraspinatus tendinopathy	Tennis, volleyball, swimming	Supraspinatus tendinopathy	Randomised controlled trial	PNE/PNS + eccentric exercise	Pain, SPADI, strength	Short-to midterm	Combined therapy effective	Limited blinding, multiple active components
**López-Royo MP, et al. 2021** ^[20]^								
Adults with patellar tendinopathy	Running, football, basketball, volleyball	Patellar tendinopathy	Randomised controlled trial	PNE+exercise vs exercise	VISA-P, pain, strength	Short-to midterm	PNE superior	Limited blinding, single centre
**Rodríguez-Huguet M, et al. 2020** ^[21]^								
Adults with supraspinatus tendinopathy	Tennis, volleyball, swimming	Supraspinatus tendinopathy	Randomised controlled trial	PNE+exercise vs exercise	Pain, SPADI	Short-to midterm	PNE superior	Limited blinding, single centre
**Rodríguez-Huguet M, et al. 2020** ^[22]^								
Adults with lateral epicondylalgia	Tennis, manual labor	Lateral epicondylalgia	Randomised controlled trial	PNE+exercise vs exercise	Pain, grip strength	Short-to midterm	PNE superior	No sham, modest sample size, single centre
**Valera-Garrido F, et al. 2019** ^[17]^								
Patients receiving PNE	Running, football, tennis	Various tendinopathies	Adverse effects study	Adverse events study	Adverse events	Short-term	Mostly mild events	No control, heterogeneous conditions
**Valera-Garrido F, et al. 2014** ^[27]^								
Adults with lateral epicondylitis	Tennis, manual labor	Chronic lateral epicondylitis	Cohort study	PNE + rehab	Pain, function	Short-to midterm	Improved outcomes	No randomised control, no blinding

DASH, Disabilities of the Arm, Shoulder and Hand; EPI, intratissue percutaneous electrolysis; PNE, percutaneous needle electrolysis; PNS, percutaneous nerve stimulation; SPADI, Shoulder Pain and Disability Index; VISA-A, Victorian Institute of Sport Assessment–Achilles; VISA-P, Victorian Institute of Sport Assessment–Patellar.

**Table 2:** Reviewed studies focusing on muscular injuries, providing details on population characteristics, intervention protocols, outcome measures, follow-up duration, and reported effects

Study population	Sport	Injury/condition	Study design	Intervention	Outcome measures	Follow up	Reported effect	Study limitations
**De-la-Cruz-Torres B, et al. 2020** ^[10]^								
Dancers	Dance	Chronic soleus injury	Randomised controlled trial	PNE + rehab	Pain, RTP	Short-term	Faster RTP	No control group, small sample, single sport
**De-la-Cruz-Torres B, et al. 2022** ^[25]^								
Female soccer players	Football	Soleus muscle injury	Pilot study	PNE + rehab	Pain, RTP	Short-term	Improved outcomes	No randomisation, no control, sport-specific
**Estévez-Rodríguez JL, et al. 2024** ^[26]^								
Professional footballers	Football	Adductor longus injury	Prospective observational cohort	Observational rehab study	RTP, performance	Season follow-up	Return to performanc e	No randomisation, potential confounding
**Gómez-Chiguano GF, et al. 2021** ^[11]^								
Mixed	Running, football, tennis, volleyball	MSK pain	Systematic review and metaanalysis	PNE for MSK pain	Pain, function	Variable	PNE effective	Many small trials, at-risk-of-bias
**Martínez-Silvan D, et al. 2022** ^[1]^								
Mixed	Running, football, tennis, volleyball	MSK injuries	Systematic review	PNE in MSK injuries	Pain, function	Variable	PNE promising	Heterogeneous designs, small studies
**Valera-Calero JA, et al. 2021** ^[30]^								
Adults with PF pain	Running, football, general fitness	PF pain syndrome	Pilot study	High vs low intensity PNE	Pain, kujala	Short-term	Both effective	Underpowered, no sham, short follow-up
**Valera-Garrido F, et al. 2020** ^[15]^								
Professional soccer players	Football	Rectus femoris injury	Cohort Study	PNE + rehab	Pain, RTP	Short-term	Good RTP	No comparator, elite athletes only

MSK, musculoskeletal; PF, patellofemoral; PNE, percutaneous needle electrolysis; RTP, return to play.

**Table 3:** PNE application protocol as described in the reviewed studies

Author	Current (mA)	Duration (s)	Deliveries (n)	Needle diameter (mm)	Device	Frequency (per week)	Average total sessions (n)
Abat F, et al. 2014 ^[5]^	3	NR	NR	0.3	EPI Advanced Medicine®	1	4.5
Arias-Buría JL, et al. 2015^[9]^	0.35	72	1	0.3	EPTE®	1	4
Valera-Garrido F, et al. 2020 ^[15]^	1–3	3	5	0.3	Physio Invasiva®	1	1
Doménech-García V, et al. 2024 ^[18]^	3	3	3	0.25	Cesmar EPI®	0.5	4
Góngora-Rodríguez J, et al. 2024 ^[19]^	0.35	72	1	0.3	EPTE®	1	4
López-Royo MP, et al. 2021 ^[20]^	3	3	3	0.25	NR	0.5	4
Rodríguez-Huguet M, et al. 2020 ^[21]^	0.35	72	1	NR	EPTE®	1	4
Rodríguez-Huguet M, et al. 2020 ^[22]^	0.35	72	1	0.3	EPTE®	1	4
Calderón-Díez L, et al. 2020^[23]^	3	10	NR	0.3	EPI Advanced Medicine®	1	5.9
De-la-Cruz-Torres B, et al. 2020 ^[10]^	2.5	3	3	0.3	Physio Invasiva®	1	2
De-la-Cruz-Torres B, et al. 2022 ^[25]^	1.5	3	3	0.3	Physio Invasiva®	1	4
Estévez-Rodríguez JL, et al. 2024 ^[26]^	1.5–2	3	3	0.3	NR	1	1
Valera-Garrido F, et al. 2014 ^[27]^	4–6	3	NR	0.3	Cesmar EPI®	1	4

EPI, intratissue percutaneous electrolysis; EPTE, Electrolisis Percutánea Terapéutica (Therapeutic Percutaneous Electrolysis); mA, milliamperes; mm, millimetres; n, number; NR, not reported; PNE, percutaneous needle electrolysis; s, seconds

## References

[1] Martínez-Silván D, Santomé-Martínez F, Champón-Chekroun AM, Velázquez-Saornil J, Gómez-Merino S, Cos-Morera MA (2022). Clinical use of percutaneous needle electrolysis in musculoskeletal injuries: a critical and systematic review of the literature. Apunts Sports Medicine.

[2] Valera Garrido F, Polidori F, Benavent Canet J, Sicarrats Botet FI, Martinez Ramirez P, Calvo S (2019). Clinical criteria for the application of percutaneous needle electrolysis in tendinopathies: An expert consensus document and cross-sectional study among physical therapists. Rev Fisioter Invasiva.

[3] Jiménez-Rubio S, Valera-Garrido F, Minaya-Muñoz F, Navandar A (2020). Ultrasound-guided percutaneous needle electrolysis and rehabilitation and reconditioning program following a hamstring injury reduces return to play time in professional soccer players: A case series. Revista Fisioterapia Invasiva.

[4] Havelin J, King T (2018). Mechanisms underlying bone and joint pain. Curr Osteoporos Rep.

[5] Abat F, Diesel WJ, Gelber PE, Polidori F, Monllau JC, Sanchez-Ibañez JM (2014). Effectiveness of the Intratissue Percutaneous Electrolysis (EPI®) technique and isoinertial eccentric exercise in the treatment of patellar tendinopathy at two years followup. Muscles Ligaments Tendons J.

[6] Peñin-Franch A, García-Vidal JA, Martínez CM, Escolar-Reina P, Martínez-Ojeda RM, Gómez AI (2022). Galvanic current activates the NLRP3 inflammasome to promote type I collagen production in tendon. Elife.

[7] Arias-Buría JL, Borrella-Andrés S, Rodríguez-Sanz J, López-de-Celis C, Malo-Urriés M, Fernández-de-Las-Peñas C (2023). Precision and safety of ultrasound-guided versus palpation-guided needle placement on the patellar tendon: a cadaveric study. Life.

[8] Abat F, Sánchez-Sánchez JL, Martín-Nogueras AM, Calvo-Arenillas JI, Yajeya J, Méndez-Sánchez R (2016). Randomised controlled trial comparing the effectiveness of the ultrasound-guided galvanic electrolysis technique (USGET) versus conventional electro-physiotherapeutic treatment on patellar tendinopathy. J Exp Orthop.

[9] Arias-Buría JL, Truyols-Domínguez S, Valero-Alcaide R, Salom-Moreno J, Atín-Arratibel MA, Fernández-de-Las-Peñas C (2015). Ultrasound-guided percutaneous electrolysis and eccentric exercises for subacromial pain syndrome: A randomised clinical trial. Evidence-Based Complementary and Alternative Medicine.

[10] De-la-Cruz-Torres B, Barrera-García-Martí I, Valera-Garrido F, Minaya-Muñoz F, Romero-Morales C (2020). Ultrasound-Guided Percutaneous Needle Electrolysis in dancers with chronic soleus injury: A randomised clinical trial. Evidence-Based Complementary and Alternative Medicine.

[11] Gómez-Chiguano GF, Navarro-Santana MJ, Cleland JA, Arias-Buría JL, Fernández-de-Las-Peñas C, Ortega-Santiago R (2021). Effectiveness of ultrasound-guided percutaneous electrolysis for musculoskeletal pain: A systematic review and meta-analysis. Pain Med.

[12] Augustyn D, Paez A (2022). The effectiveness of intratissue percutaneous electrolysis for the treatment of tendinopathy: a systematic review. S Afr J Sports Med.

[13] Abat F, Gelber PE, Polidori F, Monllau JC, Sanchez-Ibañez JM (2015). Clinical results after ultrasound-guided intratissue percutaneous electrolysis (EPI®) and eccentric exercise in the treatment of patellar tendinopathy. Knee Surgery, Sports Traumatology, Arthroscopy.

[14] Moreno C, Mattiussi G, Nunez FJ, Messina G, Rejc E (2017). Intratissue percutaneous electrolysis combined with active physical therapy for the treatment of adductor longus enthesopathy-related groin pain: a randomised trial. J Sports Med Phys Fitness.

[15] Valera-Garrido F, Jiménez-Rubio S, Minaya-Muñoz F, Estevez-Rodriguez JL, Navandar A (2020). Ultrasound-guided percutaneous needle electrolysis and rehab and reconditioning program for rectus femoris muscle injuries: a cohort study with professional soccer players and a 20-week follow-up. Appl Sci.

[16] Margalef R, Bosque M, Minaya-Muñoz F, Valera-Garrido F, Santafe MM (2021). Safety analysis of percutaneous needle electrolysis: a study of needle composition, morphology, and electrical resistance. Acupunct Med.

[17] Valera-Garrido F, Minaya Muñoz F, Ramírez Martínez P, Medina-Mirapeix F (2019). Adverse effects associated to the application of ultrasound-guided percutaneous needle electrolysis. Revista Fisioterapia Invasiva.

[18] Doménech-García V, Pecos-Martín D, Blasco-Abadía J, Bellosta-López P, López-Royo MP (2024). Placebo and nocebo effects of percutaneous needle electrolysis and dry-needling: an intra and inter-treatment sessions analysis of a three-arm randomised double-blinded controlled trial in patients with patellar tendinopathy. Front Med.

[19] Góngora-Rodríguez J, Rosety-Rodríguez MÁ, Rodríguez-Almagro D, Martín-Valero R, Góngora-Rodríguez P, Rodríguez-Huguet M (2024). Structural and functional changes in supraspinatus tendinopathy through percutaneous electrolysis, percutaneous peripheral nerve stimulation and eccentric exercise combined therapy: A single-blinded randomised clinical trial. Biomedicines.

[20] López-Royo MP, Ríos-Díaz J, Galán-Díaz RM, Herrero P, Gómez-Trullén EM (2021). A comparative study of treatment interventions for patellar tendinopathy: a randomised controlled trial. Arch Phys Med Rehabil.

[21] Rodríguez-Huguet M, Góngora-Rodríguez J, Rodríguez-Huguet P, Ibañez-Vera AJ, Rodríguez-Almagro D, Martín-Valero R (2020). Effectiveness of percutaneous electrolysis in supraspinatus tendinopathy: a single-blinded randomised controlled trial. J Clin Med.

[22] Rodríguez-Huguet M, Góngora-Rodríguez J, Lomas-Vega R, Martín-Valero R, Díaz-Fernández Á, Obrero-Gaitán E (2020). Percutaneous electrolysis in the treatment of lateral epicondylalgia: A single-blind randomised controlled trial. J Clin Med.

[23] Calderón-Díez L, Sánchez-Sánchez JL, Belón-Pérez P, Sánchez-Ibáñez JM (2020). Prospective analysis of the beneficial effects of intratissue percutaneous electrolysis (EPI) combined with eccentric exercise in the treatment of chronic Achilles tendinopathy. J Orthop Res Ther.

[24] De-la-Cruz-Torres B, Albornoz Cabello M, Garcia Bermejo P, Naranjo Orellana J (2016). Autonomic responses to ultrasound-guided percutaneous needle electrolysis of the patellar tendon in healthy male footballers. Acupunct Med.

[25] De-la-Cruz-Torres B, Romero-Rodríguez B, Romero-Morales C (2022). Ultrasound-guided percutaneous needle electrolysis combined with therapeutic exercise may add benefit in the management of soleus injury in female soccer players: a pilot study. J Sport Rehabil.

[26] Estévez-Rodríguez JL, Rivilla-García J, Jiménez-Rubio S (2024). Is it possible to improve performance in competition after an adductor longus injury in professional football players?. J Sport Rehabil.

[27] Valera-Garrido F, Minaya-Muñoz F, Medina-Mirapeix F (2014). Ultrasound-guided percutaneous needle electrolysis in chronic lateral epicondylitis: short-term and long-term results. Acupunct Med.

[28] McKay AKA, Stellingwerff T, Smith ES, Martin DT, Mujika I, Goosey-Tolfrey VL (2021). Defining training and performance caliber: a participant classification framework. Int J Sports Physiol Perform.

[29] López-Royo MP, Gómez-Trullén EM, Ortiz-Lucas M, Galán-Díaz RM, Bataller-Cervero AV, Al-Boloushi Z (2020). Comparative study of treatment interventions for patellar tendinopathy: a protocol for a randomised controlled trial. BMJ Open.

[30] Valera-Calero JA, Sánchez-Mayoral-Martín A, Varol U (2021). Short-term effectiveness of high and low-intensity percutaneous electrolysis in patients with patellofemoral pain syndrome: A pilot study. World J Orthop.

[31] Lorenz J, Hauck M, Paur RC, Nakamura Y, Zimmermann R, Bromm B (2005). Cortical correlates of false expectations during pain intensity judgments: A possible manifestation of placebo/nocebo cognitions. Brain Behav Immun.

[32] Petersen GL, Finnerup NB, Colloca L, Amanzio M, Price DD, Jensen TS (2014). The magnitude of nocebo effects in pain: a meta-analysis. Pain.

[33] Laramée A, Léonard G, Morin M, Roch M, Gaudreault N (2021). Neurophysiological and psychophysical effects of dry versus sham needling of the infraspinatus muscle in patients with chronic shoulder pain: A randomised feasibility study. Arch Physiother.

[34] Fakontis C, Iakovidis P, Lytras D, Kasimis K, Koutras G, Ntinou SR (2023). Efficacy of percutaneous needle electrolysis versus dry needling in musculoskeletal pain: A systematic review and meta-analysis. J Back Musculoskelet Rehabil.

[35] Sánchez-Sánchez JL, Calderón-Díez L, Herrero-Turrión J, Méndez-Sánchez R, Arias-Buría JL, Fernández-de-Las-Peñas C (2020). Changes in gene expression associated with collagen regeneration and remodeling of extracellular matrix after percutaneous electrolysis on collagenase-induced Achilles tendinopathy in an experimental animal model: A pilot study. J Clin Med.

[36] Arita A, Tobita M (2024). Adverse events related to platelet-rich plasma therapy and future issues to be resolved. Regen Ther.

[37] Coombes BK, Bisset L, Vincenzino B (2010). Efficacy and safety of corticosteroid injections and other injections for management of tendinopathy: A systematic review of randomised controlled trials. Lancet.

[38] de Miguel Valtierra L, Moreno JS, Fernández-de-Las-Peñas C, Cleland JA, Arias-Buría JL (2018). Ultrasound-guided application of percutaneous electrolysis as an adjunct to exercise and manual therapy for subacromial pain syndrome: A randomised clinical trial. J Pain.

[39] Marieswaran M, Jain I, Garg B, Sharma V, Kalyanasundaram D (2018). A review on biomechanics of anterior cruciate ligament and materials for reconstruction. Appl Bionics Biomech.

[40] Binder-Markey BI, Broda NM, Lieber RL (2020). Intramuscular anatomy drives collagen content variation within and between muscles. Front Physiol.

[41] Margalef R, Valera-Garrido F, Minaya-Muñoz F, Bosque M, Ortiz N, Santafe MM (2020). Percutaneous needle electrolysis reverses neurographic signs of nerve entrapment by induced fibrosis in mice. Evid Based Complement Alternat Med.

